# *In Vitro* Anticancer Potential of *Berberis lycium* Royle Extracts against Human Hepatocarcinoma (HepG2) Cells

**DOI:** 10.1155/2020/8256809

**Published:** 2020-10-14

**Authors:** Kiren Mustafa, Hassan Mohamed, Aabid Manzoor Shah, Shaoxuan Yu, Muhammad Akhlaq, Haifang Xiao, Shaoqi Li, Tahira Naz, Shaista Nosheen, Xueyuan Bai, Yuanda Song

**Affiliations:** ^1^Colin Ratledge Center of Microbial Lipids, Shandong University of Technology, School of Agriculture Engineering and Food Science, Zibo 255000, China; ^2^Department of Botany and Microbiology, Faculty of Science, Al-Azhar University, Assiut 71524, Egypt; ^3^PMAS-University of Arid Agriculture, Rawalpindi, Pakistan

## Abstract

Human liver cancer has emerged as a serious health concern in the world, associated with poorly available therapies. The *Berberis* genus contains vital medicinal plants with miraculous healing properties and a wide range of bioactivities. In this study, different crude extracts of *B. lycium* Royle were prepared and screened against Human Hepatocarcinoma (HepG2) cell lines. The water/ethanolic extract of *B. lycium* Royle (BLE) exhibited significant antiproliferative activity against the HepG2 cancer cell line with an IC_50_ value of 47 *μ*g/mL. The extract decreased the clonogenic potential of HepG2 cells in a dose-dependent manner. It induced apoptotic cell death in HepG2 cells that were confirmed by cytometric analysis and microscopic examination of cellular morphology through DAPI-stained cells. Biochemical evidence of apoptosis came from elevating the intracellular ROS level that was accompanied by the loss of mitochondrial membrane potential. The mechanism of apoptosis was further confirmed by gene expression analysis using RT-qPCR that revealed the decline in Bcl-2 independent of p53 mRNA and a rise in CDK1 while downregulating CDK5, CDK9, and CDK10 mRNA levels at 48 h of BLE treatment. The most active fraction was subjected to HPLC which indicated the presence of berberine (48 *μ*g/mL) and benzoic acid (15.8 *μ*g/mL) as major compounds in BLE and a trace amount of luteolin, rutin, and gallic acid. Our study highlighted the importance of the most active BLE extract as an excellent source of nutraceuticals against Human Hepatocarcinoma that can serve as an herbal natural cure against liver cancer.

## 1. Introduction

Hepatocellular carcinoma (HCC) is a primary liver cancer, reported as the leading cause of cancer-associated deaths due to poor prognosis [1]. HCC is annually diagnosed in more than 6 million people and accounts for more than 80% of liver cancer cases [1]. Currently, there is no clinically proven effective therapy for advanced liver cancer patients [1, 2]. Due to the large scale development of acquired or intrinsic chemoresistance, the majority of HCC patients do not respond to available chemotherapies [3]. Thus, the development of effective and novel therapies is of utmost priority to combat such a devastating disease [2, 3]. Plants are known as important sources of new chemical entities suitable for anticancer drug discovery and development, and many plant species are already being used to treat or prevent the development of cancer. Multiple researchers have identified different species of plants that have demonstrated anticancer properties with a lot of focus on those that have been used in herbal medicine in developing countries. The *Berberis* genus contains vital medicinal plants with miraculous healing properties and a wide range of bioactivities [4, 5]. The *Berberis* species are part of British and Indian pharmacology. *B. lycium* Royle is found at high altitudes in Pakistan and is commonly used in folk medicine against ulcer, diabetes, skin diseases, and jaundice as well as for treating muscular dystrophy and rheumatism [6, 7]. *B. lycium* is an erect, small, rigid shrub about 1.0–2.5 m tall, with a thick woody shoot covered with thin brittle bark. Since ancient times, the *Berberis* species have been used in Unani, Ayurvedic, and Chinese traditional medicines [4].

*B. lycium* roots form a reputed drug in the Ayurvedic medicines; the root contains several alkaloids, out of which the most prominent one is berberine, which is universally present in the rhizomes of the *Berberis* species and finds its application in the preparation of drugs against cancer [8]. Several *Berberis* species and their bioactive compounds possess a broad series of pharmacological activities like anti-inflammatory, antitumor, antipyretic and antidiarrheal, antimicrobial, and antiarrhythmic activities [4]. A critical evaluation revealed that pharmacological studies in the past have a deficiency in the identification of bioactive constituents responsible for pharmacological effects [9]. *B. lycium* is also of great importance as a household remedy for the treatment of various diseases and was the main focus of previous research against cancer [8, 10].

Keeping in view the therapeutic effect of *B. lycium*, the roots of the plants were subjected against hepatocellular carcinoma (HepG2) cells and showed moderate activity. Moreover, little research has been done on elucidating the molecular mechanism of either berberine or *B. lycium* crude extract in HepG2 cells. Recently, a study on berberine from *Coptis chinensis* Franch revealed the involvement of the mitochondria-mediated apoptosis pathway in Huh7 liver cancer cells via the caspase cascade [11]. Furthermore, in past studies, the inhibitory effect of the *Berberis* plant extract and berberine compound was revealed in HL-60 cells by the arrest of the cells in the S phase [12]; another study revealed an anticancer effect of commercially available berberine compound on liver cancer cells via the AMPK-mediated mitochondrial pathway [13]. But as berberine alone is not as safe as the crude extract, the need to investigate the most active and at the same time safe extract of *B. lycium* against HepG2 cells is urged. Furthermore, the role of cell cycle arrest markers or involvement of mitochondria in the cell death process has not been investigated previously for *B. lycium* Royle.

Based on previous research gaps, the current study was aimed at finding the most effective crude extract from *B. lycium* Royle plant against the liver (HepG2) cancer cell line for the first time. We also aimed to identify phenolic compounds and alkaloids in the most active crude extract as well as elucidate the molecular details about cell cycle arrest and apoptosis inside the HepG2 cells.

## 2. Materials and Methods

### 2.1. Extraction of Plant Material

*B. lycium* Royle plants were collected from high altitudes of the Murree hills in Pakistan. Identification of the plant was confirmed by the Department of Agriculture Engineering and Food Science, Shandong University of Technology, China. The plant material was shade dried and ground into powder. One-gram powder of plant material was added in 40 mL of respective solvents (Acetone, water, methanol, 50% ethanol, ethyl acetate, hexane, and n-butanol) and left for 2 to 3 days. The obtained plant solvent mixture was first filtered through filter paper (Aladdin Shanghai Biochemical Technology, Co., Ltd.), and the filtered solvent was concentrated in a rotary evaporator (model RE-201D Henan, China) and oven-dried at 30°C. The yield was calculated as the weight of the extract/weight of dissolved plant powder × 100. The acquired extracts were stored at −80 °C until used in experimental assays.

### 2.2. Cytotoxicity Assay

The HepG2 and HUVEC cells were attained from the Kunming Institute of Zoology, Chinese Academy of Sciences (Kunming, China). Both of the cells were cultured in DMEM (Dulbecco's modified Eagle's medium) (Thermo Fisher Scientific, USA) supplemented with 10% FBS (Foetal Bovine Serum) (Gibco, NY, USA) and 1% of both antibiotics, penicillin (100 U/mL), and streptomycin (100 mg/mL) in an incubator at 37°C humidified with 5% CO_2_. The cytotoxic effect of *B. lycium* extracts on HepG2 and HUVECs was determined by MTT assay [14, 15]. Briefly, the cells from the exponential phase were grown in an incubator at 37°C and 5% CO_2_ in a 96-well plate (Corning, China) overnight, followed by addition of various solvent-based extracts, e.g., water, butanol, acetone, ethanol, and ethyl acetate extracts for 48 h. Organic extracts were redissolved in DMSO completely as a stock solution so that *DMSO* < 1% on final dilutions of BL extracts with DMEM media. Thus, serial dilutions of BL extracts were prepared, starting from 0.0625 mg/mL to highest 8 mg/mL with DMEM media to apply on HepG2 cells. After a period of incubation, MTT solution (20 *μ*L or 5 mg/mL) along with 80 *μ*L of DMEM media was added per well followed by reincubation for 4 h at 37°C in darkness. Then, MTT dye was replaced with 100 *μ*L of DMSO per well. The OD was analyzed on a Bio-Rad 680 microplate reader (Bio-Rad Laboratories, Hercules, CA, USA) at 570 nm wavelength. Each experiment was repeated three times, and each dilution was put on a 96-well plate in five replications.

### 2.3. Staining with Acridine Orange (AO) and Ethidium Bromide (EB)

To observe the apoptosis in cancer cells, nucleic acid stains like AO and EB (Cat # E607308, Sangon Biotech (Shanghai) Co., Ltd.) were used. The cells were grown on a 96-well plate (Corning, China) for 24 h preceded by incubation with BLE (4, 2, 1, and 0.5 mg/mL) extract for 24 h. The standard manual provided with the kit was followed. Briefly, we diluted 10x buffers with distilled sterile water to 1x. 5 *μ*L of each AO, and EB was added into 90 *μ*L of 1x buffer. We removed the BLE extract after completion of a 24 h period of incubation from the 96-well plate. The extract residues were washed from the surface of cells on the 96-well plate with PBS two times, followed by homogenous staining with 100 *μ*L of AO/EB suspension for 15 min. at 37°C in the dark. Subsequently, the added stain was replaced by PBS to wash extra dye, and the cells were examined under the fluorescence microscope (Zeiss, Oberkochen, Germany) at two wavelengths (excitation: 500 nm, and emission: 526 nm) for DNA. The AO gives a green color to the cells, and EB stains only necrotic and apoptotic cells that appear orange or with weak/blurred fluorescence.

### 2.4. DAPI (4′,6-Diamidino-2-Phenylindole) Staining

The nucleic acid of control and BLE-treated HepG2 were stained by DAPI (C1005, Beyotime) following the manual's guide. To be precise, the cells were cultured on a 6-well plate at 37°C in an incubator supplied with 5% CO_2_ for overnight followed by inoculation of BLE at 4, 2, 1, and 0.5 mg/mL for 24 h. After incubation, the wells were washed with PBS carefully without aspiring the cells and added with 25 *μ*g/mL DAPI in PBS (5 *μ*L DAPI in 995 mL of PBS) proceeded by incubation in the dark for 20 min. Subsequently, excess stain from the wells was washed with PBS and observed under the fluorescence microscope (Zeiss, Oberkochen, Germany).

### 2.5. Colony Formation Assay

The anticolonization ability of BLE against cancer cells was analyzed by the colony-forming assay as mentioned in previous studies [16] with minor changes. Cancer cells were plated on a 6-well plate at a density of 1.0 × 10^4^ cells/well for one day. BLE was added at different concentrations (control, 0.25, 0.5, 1, 2, and 4 mg/mL) on the cells, and it was incubated for 24 h. After the incubation period, BLE-containing media was removed, and the cells were washed with PBS carefully followed by incubation of these cells with the complete media containing 10% FBS and 1% pen-strep for 8 more days to analyze anticolonization potential of BLE. After every four days, complete media from cultured cells was replaced with new complete media. Subsequently, the cells were washed with PBS two times followed by staining with crystal violet stain (C0121, Beyotime, China) for 10 min. and again washed three times with PBS and air-dried. Finally, photos were taken with a digital camera.

### 2.6. Reactive Oxygen Species Assay

To analyze the stress induction in liver cancer cells by BLE extract and to assess whether the death induction ability of BLE extract depends on ROS generation, we used DCFH-DA (S0033, Beyotime). Intracellular esterase hydrolyzes the DCFH-DA into DCFH, which can react with H_2_O_2_ to generate free radicals, e.g., DCF^·^ inside the cells. The HepG2 cells were inoculated with (0.25, 0.5, 1, 2, and 4 mg/mL) concentrations of BLE for 24 h once they formed a 70-80% confluence layer. The manual's guide along with the kit was followed for this assay. Therefore, after incubation of cells with BLE for 24 h, 10 *μ*M DCFH-DA was prepared in PBS and 100 *μ*L was added in each well of the 96-well plate. The plates were incubated away from light at 37°C for 30 min. The wells were drained off with PBS after incubation with the reagent to remove the extracellular dye. It was examined immediately under the fluorescence microplate reader at 488 nm excitation and 525 nm emission wavelengths. Meanwhile, the plates were observed under the fluorescence microscope as well.

### 2.7. Mitochondrial Membrane Potential (*ψ*) Assessment by JC-1 Probe

Mitochondria membrane damage was observed by a lipophilic fluorescent dye JC-1 (5,5′,6,6′-tetrachloro-1,1′,3,3′-tetraethyl benzimidazolyl carbocyanine iodide) (Catalog No. C2005, Beyotime, China) as cited in previous protocols [16]. The actively replicating cells were cultured on a 96-well plate (1 × 10^5^ cells/well) overnight, followed by inoculation with BLE (0.25–4 mg/mL) extract for 24 h. It was followed by the replacement of the BLE extract with 5 *μ*g/mL of the JC-1 probe for 30 min in the dark at 37°C. The cells were washed twice with PBS before examination under the fluorescent microscope/microplate reader. The fluorescence microplate reader was used to measure the fluorescence intensities at two wavelengths (excitation: 485 nm, emission: 535 nm) for the JC-1 monomeric form, while excitation *λ*: 535 nm and emission *λ*: 590 nm for J-aggregates. The values were expressed in % of ratio relative to control (OD red/OD green) × 100.

### 2.8. Cell Cycle Analysis by Flow Cytometry

HepG2 cells cultured on tissue culture (10 mm^3^) plates which were incubated with BLE extract at the lowest, medium, and highest concentrations (0.0625, 0.5, and 1.0 mg/mL) for 24 h and subsequently stained by a cell cycle and apoptosis detection kit (C1052, Beyotime, China) by following the manufacturer's instructions. The accumulation of cells at various phases of the cell was analyzed using flow cytometry (BD FACSCalibur, Becton Dickinson Medical Devices Shanghai Co., Ltd.). The CellQuest and ModFit LT model of analysis were used to measure total cell events, cellular debris, and cell cycle (G0/G_1_, G_2_, and S phase) arrest/ploidy modes. The experiment was repeated three times.

### 2.9. RT-qPCR (Quantitative Real-Time Polymerase Chain Reaction)

HepG2 cells were incubated with 1.0 mg/mL of BLE for 24, 48, and 72 h along with an untreated group on 10 mm^3^ culture plates. After the respective time of treatments, the total RNA was extracted with a TRIzol total RNA extractor (B511311, Sangon Biotech) by following the manufacturer's protocol. Briefly, cells were washed two times with chilled PBS while placing on ice, followed by the addition of 1.0 mL TRIzol, and cells were allowed to stay for 10 min. at room temperature, preceded by phase separation with chloroform, precipitation of RNA with isopropanol, and washing the extracted RNA with 75% ethanol and subsequent drying of RNA pellet. Completely dried RNA was suspended in RNAase-free water. The concentration and purity of RNA were determined by nanodrop and by 1% agarose gel electrophoresis. The original RNA was stored at −80°C. First-strand cDNA was synthesized from 1 *μ*g RNA by a RevertAid First Strand cDNA Synthesis Kit (K1622, Thermo Scientific) using oligo(dT) primers according to manual instructions, followed by PCR amplification of the first cDNA by using GAPDH primers provided with the kit and Premix Taq Polymerase enzyme. The complementary DNA was selectively amplified for mitochondrial regulatory apoptotic marker genes (Bcl-2, TP53) and cell cycle (CDKs) genes by using a Heifi™ qPCR SYBR® Green Master Mix reverse transcription polymerase chain reaction premixture (11201ES60) on a LightCycler® 480. The genes and sequence of primers are given in the Supplementary Table (available here). The primer efficiency was first checked and validated before proceeding for each gene expression assessment. Both the *β*-actin and 18S were used as internal reference genes initially for RT-qPCR for untreated HepG2 cells. Subsequently, based on consistent results, *β*-actin was used as the only internal housekeeping gene for BLE-treated cells. For statistical analysis of target gene and reference gene expression, the standalone software REST 2009 was used with efficiency correction. *ΔΔ*ct calculation was used to calculate the target gene expression.

### 2.10. Phytochemical Analysis

#### 2.10.1. Thin Layer Chromatography (TLC) Analysis

For qualitative and partial quantitative detection of constituents of three extracts such as BLE, BA, and B-H_2_O extracts, TLC was carried out. For TLC, the solvent system was used as described previously [12] with minor modification as toluene : ethyl acetate : methanol : isopropyl alcohol : H_2_O : hexane in a ratio of 12 : 6 : 2.5 : 2.5.0.5 : 0.5. The TLC chamber was saturated with the above solvent system prior to placement of the TLC plate in it. The alkaloids (berberine and palmatine) were detected under a UV scanner at short and long wavelengths.

#### 2.10.2. High Pressure Liquid Chromatography (HPLC) of BLE

The freshly prepared BLE extract was analyzed for total phenolic contents in terms of gallic acid by a Folin-Ciocalteu reagent [17] and total flavonoid contents in terms of a quercetin equivalent per mg of a plant by aluminum chloride-based colorimetric assay [18] with minor modifications. The HPLC was done for identification and quantification of phenolic compounds by following protocol [19] with minor modifications. HPLC grade standards (chlorogenic acid, apigenin, resveratrol, luteolin, gallic acid, *β*-sitosterol and rutin, benzoic acid, and quercetin) and alkaloids palmatine and berberine chloride were purchased from Sigma Chemical Co. (USA), and HPLC grade solvents such as acetone, methanol, water, and acetic acid were purchased from Merck (China). HPLC coupled with a diode array detector (Agilent Technologies) was used with a 5 cm flow cell, automatic sample injection valve equipped with a 20 *μ*L loop, and CDC system manager as a data processor. The separation was achieved by an InfinityLab Poroshell 120 EC-C18 column (4.6 *mm* × 150 *mm*, 4 *μ*m particle size, Agilent Technologies, USA). The stock solutions of 1.0 mg/mL for the mentioned standards were prepared as described in the protocol with minor modifications. The mobile phase is made up of 1% aq. acetic acid solution (solvent A) and acetonitrile (solvent B) with a flow rate of 1 mL/min. The gradient elution was performed by changing the proportion of solvent B to solvent A as 10%-40% for 15 min., from 40 to 60% solvent B in 25 min. and from 60 to 90% solvent B in 30 min. The composition of the solvent running in the last 10 minutes was as Solvent A : Solvent B (90 : 10). The total analysis time per sample was 40 min. The chromatograms for HPLC standards and samples were detected at 272 nm wavelength. The retention time for each standard was noted after an individual run and each standard stock solution was diluted to 20, 40, 60, and 80 *μ*g/mL concentrations. Then, a mixture of mentioned standards was prepared and analyzed by HPLC, and the standard curve was plotted between area versus concentration of standards, where the correlation coefficient (*R*^2^ > 0.99) reveals the measure of linearity. Hence, the quality and quantity of different phenolic/flavonoid/alkaloid content were determined in the BLE extract.

### 2.11. Statistical Analysis

All data were presented as mean ± SD. One-way analysis of variance (ANOVA) was used to determine the level of significance among various groups. For statistical analysis and probit regression analysis at a 95% confidence interval, IBM SPSS statistical software package (version 17) was used. A probability value of *p* < 0.05 (∗) was considered as statistically significant.

## 3. Results

### 3.1. BL Extracts Induced Cytotoxicity

**Figure 1:**
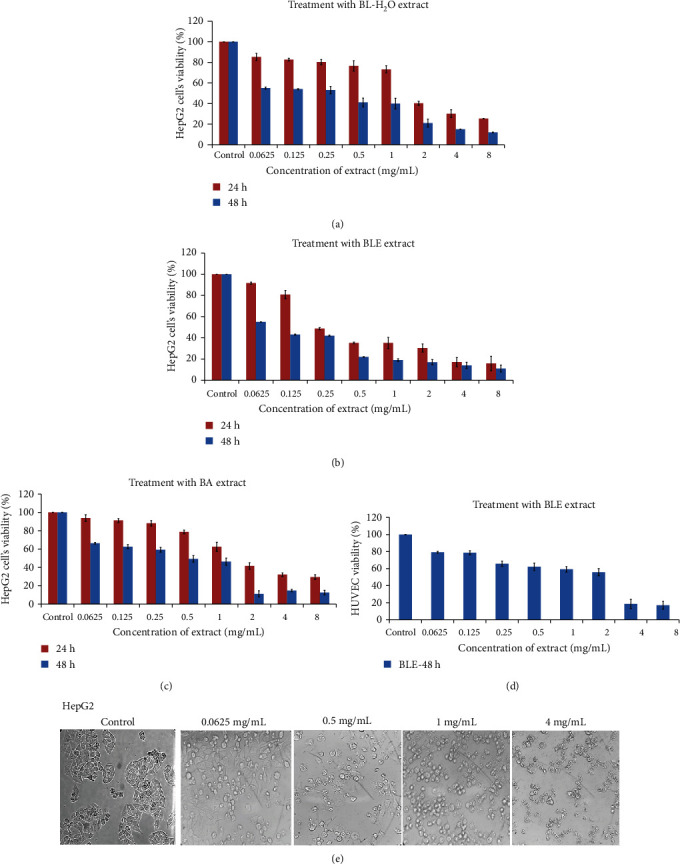
Time- and dose-dependent inhibitory effect of *B. Lycium* Royle extracts on HepG2 cells and HUVECs. (a) Water extract on the cell's viability for 24 and 48 hours. (b) Effect of 50% ethanol extract of BLE on HepG2 cell's growth for 24 and 48 h. (c) BL acetone extract decreased the viability of HepG2 cells in a dose- and time-dependent manner. (d) Inhibitory effect of BL water extract on HUVECs as observed for a maximum of 48 h. (e) Morphological examination of HepG2 cells after 24 h of treatment with BL-50% ethanol extract, with a light microscope (only a few photographs have been shown here with a significant difference). All values are presented as mean ± SD from three independent experiments.

In this study, different solvents were used for the preparation of *B. lycium* root extracts and the extraction results showed that yield was the highest in water and lowest in butanol as shown in Table 1. As a result of initial cytotoxic screening of different BL extracts against HepG2 cells for 48 h of incubation, it was found that 50% ethanol-based crude extract (BLE) was the most effective (IC_50_ value of 0.047 mg) extract followed by other extracts as shown in Table 1. Thus, we proceeded with the detailed study on BLE against HepG2 cells. Based on the results of the initial preliminary screening on HepG2 cells, we have chosen BE, BA, and BW extracts to check the effect of dose and time on the survival of HepG2 cells. Thus, we found both the time- and dose-dependent cytotoxic potentials of three extracts as depicted in Figure 1(a). On HUVECs, we tested only the most effective BLE extract for the maximum time (48 h) of treatment and found an IC_50_ value of 1.200 mg/mL. This result shows that our extract is more toxic to the liver cancer cell line as compared to normal cells. The strong cytotoxic effect of the BLE extract was also observed by a light-inverted microscope as shown in Figure 1(b); it revealed the formation of round apoptotic bodies and clear membrane fragmentation/disruption.

### 3.2. AO/EB and DAPI Staining and Anticolonization Potential of BLE against HepG2 Cells

**Figure 2:**
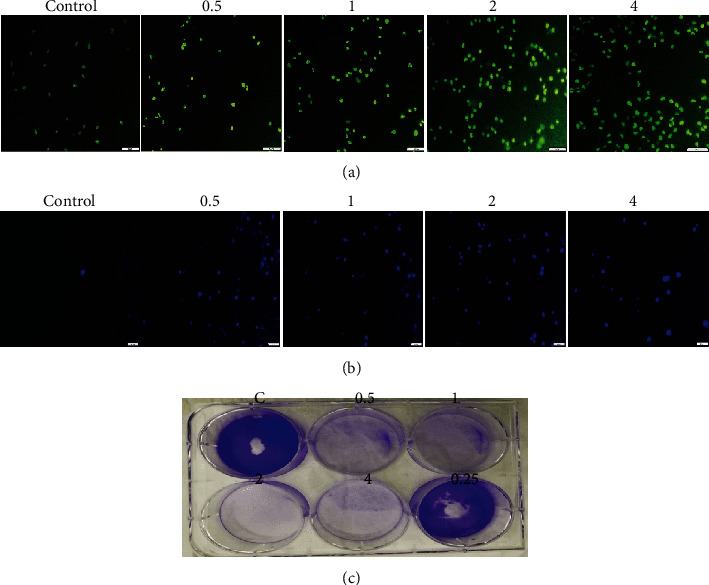
Fluorescent microscopy of HepG2 cells and colony formation assay. (a) AO/EB staining of HepG2 cells after incubation with 0.5, 1, 2, and 4 mg/mL of BLE for 24 h. Live cells stained as uniformly light green in control (untreated) cells, while early apoptotic cells stained as green cells with bright green nuclei that gives the bright green appearance, and necrotic cells/late apoptotic cells stained as light orange. (b) DAPI staining shows the control (untreated) cells as uniformly light blue and the cells after 24 hour of incubation with BLE appeared bright blue with dense nuclei due to chromosomal condensation and nuclear fragmentation as a result of apoptosis. All pictures were taken under the fluorescence microscope (scale bar = 20 *μ*m). (c) The HepG2 cells were grown on a 6-well plate and subsequently incubated with BLE for 24 h, followed by 8-day growth in complete media; it exhibited complete disassociation of colony-forming potential of cancer cells at all high (4, 2, 1, and 0.5 mg/mL) doses and its ability diminishes at 0.25 mg/mL.

We used acridine orange (AO) and ethidium bromide (EB) dyes to observe the apoptosis of cancer cells after treatment with BLE for 24 h. AO and EB are both nucleic acid staining dyes, and AO stains both viable and nonviable cells, giving a consistent green appearance to live cells without bright fluorescence in nuclei (Figure 2(a)), while EB stains only the dead cells; thus, they appear weakly orange in color or sometimes blurred. As shown in Figure 2(a), the cells at the early stage of apoptosis appear uniform green in color, and they also contain small fluorescent dots inside the cells as a result of condensation of chromosomes. The cells at the late stage of apoptosis and the cells undergoing necrosis were stained light orange as indicated by light orange stained cells at a concentration of 2 and 4 mg/mL in Figure 2(a).

Furthermore, to observe the changes in the nucleus, we stained HepG2 cells with DAPI either untreated or treated with the same concentration of BLE as we did for AO/EB. After 24 h of incubation, we found the fragmentation of nuclei as depicted by brighter fluorescence intensity at different doses of BLE as compared to the control group of the cells (Figure 2(b)).

### 3.3. Reactive Oxygen Species Generation and Mitochondrial Membrane Potential Changes

**Figure 3:**
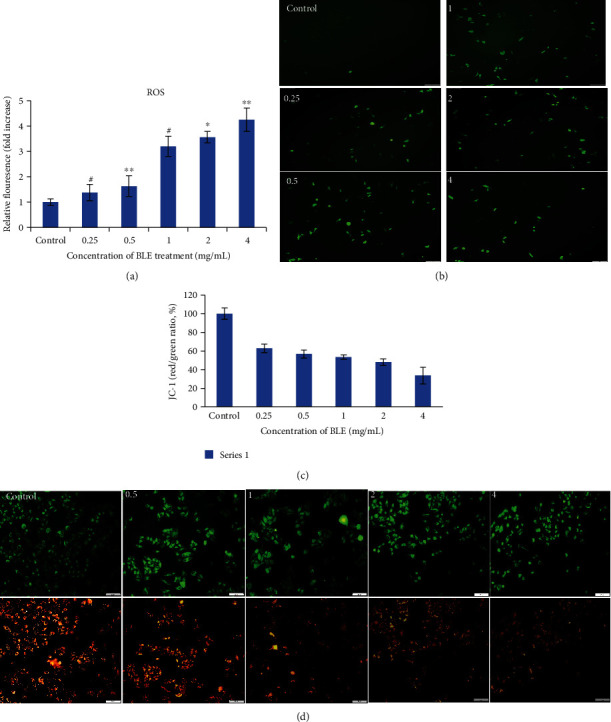
Effect of BLE on ROS generation and mitochondrial membrane potential changes in HepG2 cells. HepG2 cells were incubated without or with BLE at specified concentrations for 24 hours. (a) The generation of ROS was analyzed by DCFH-DA (10 *μ*M/L) assay in HepG2 cells, and the fluorescence was measured at excitation and emission *λ* of 488 and 525 nm, respectively, with the help of a fluorescence microplate reader. (b) Fluorescence microscopic view of HepG2 cells after ROS assay indicates an increase in green fluorescence spots due to ROS generation with BLE treatment as compared to control. (c) The bar graph represents the fluorescent intensity ratios between green fluorescence (for loss of membrane potential at 485 nm-535 nm *λ*) and red fluorescence (for normal changes in mitochondrial membrane potential at 535 nm-590 nm *λ*) by the fluorescent microplate reader. The JC-1 red fluorescence intensity was selected as 100%, and fluorescence intensity of treated samples was measured relative to control as (treated/control) × 100. (d) Florescent microscopic view of HepG2 cells for *Δψ* after staining in the dark with a JC-1 probe for 30 min and red fluorescence was present in cells with high *ψ* and vice versa for green fluorescence; photographs were taken at two *λ* (green and red). All values are mean ± SD from three independent experiments. Significant differences as compared to control were depicted by ^∗^*p*, 0.05; ^∗∗^*p*, 0.01; or ^∗∗∗^*p*, 0.001.

The reactive oxygen species act as a second messenger in death signal transduction in response to stress. We analyzed the ROS generation by DCFH-DA assay in HepG2 cells and found a significant increase (1.5-folds) in the content of reactive oxygen species inside the cancer cells at 0.5 mg/mL and 3.5- and 4-folds at 2 and 4 mg/mL of BLE treatment, respectively (Figure 3(a)). The fluorescence microscopic view of the treated cells strongly agrees with the ROS generation assay by a fluorescent microplate reader (Figure 3(b)). In order to further unveil the effect of BLE treatment on MMP of HepG2 cells, we measured the MMP by JC-1 probe assay. As JC-1 is a ratiometric dye, it forms red aggregates in the cells with high MMP and produces green monomers in the cells with low MMP. We found a significant reduction in red fluorescence and an increase in green fluorescence in a dose-dependent way for 24 h of BLE treatment, as revealed absolutely by a fluorescence microplate reader (Figure 3(c)) and by fluorescence microscope view at two wavelengths (Figure 3(d)). The % of the red/green ratio declined by 60% to 30% from 0.25 mg/mL to 4 mg/mL of BLE treatment, thus indicating a decline in MMP.

### 3.4. Cell Cycle Changes by BLE Treatment

**Figure 4:**
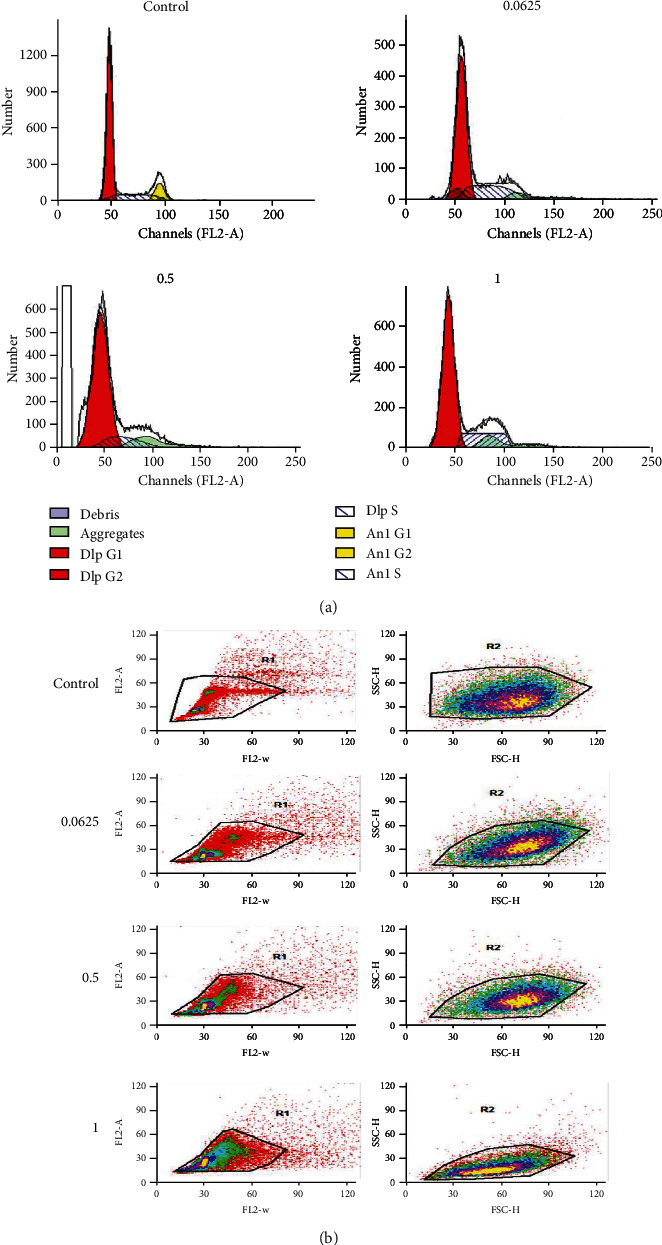
Selective arrest of the cell cycle by BLE against HepG2 cells after 24 h of incubation. (a) The histograms were made by analysis on a ModFit LT model of analysis for control and BLE-treated HepG2 cells at mentioned concentrations for 24 h. BLE arrested the HepG2 cell's cycle at the synthesis (S) phase of growth at 0.0625 mg/mL and G_1_ phase after treatment at high concentrations of 0.5 and 1 mg/mL. (b) Gate 1 is plotted with FL2-W (width) and FL2-A (area) of the scattered cells, and gate 2 defines FSC-H (forward scatter) and SSC-H (side scatter) of the stained HepG2 cells.

The HepG2 cells upon treatment with BLE extract for 24 h exhibited a different pattern of cell cycle distribution at various concentrations. As shown in Figures 4(a) and 4(b), at a low concentration (0.0625 mg/mL), BLE arrested the cell cycle at the S phase with an accumulation of 29.9% cells in the S phase. While at 0.5 mg/mL and 1 mg/mL, the same extract exhibited entirely different behavior as it caused the increase in the number of cells in the G_1_ phase. As shown in Supplementary Figure S1, 82% and 88% cells accumulated in the G_1_ phase for 0.5 and 1 mg/mL treatments, respectively, as compared to 68% cells in untreated HepG2 cells.

### 3.5. Gene Expression Changes in HepG2 Cells by BLE Treatment

**Figure 5:**
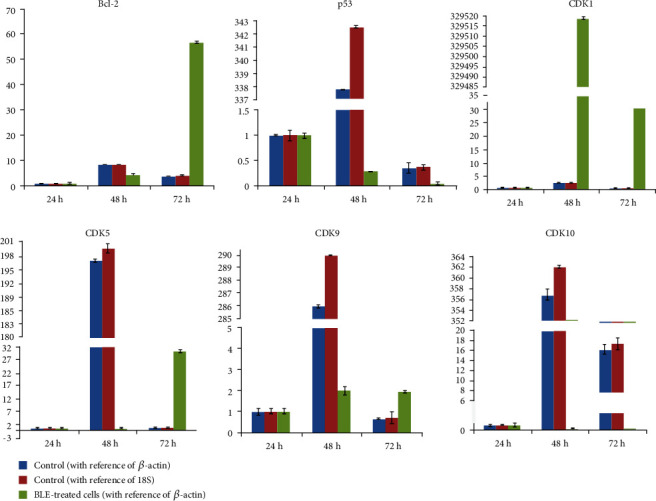
Variable genetic expression of key apoptotic/cell cycle markers after treatment with BLE. The mRNA expression is relative to 24 h. All results are stated as mean ± SD from experiments repeated three times.

We analyzed the expression of a few key genes, which play an important role in regulating mitochondrial membrane potential and cell cycle. The primer sequence for all genes is provided in Supplementary Table S1. RT-qPCR revealed the results of control/untreated HepG2 cells and BLE-treated cells at 1 mg/mL dose for 24, 48, and 72 h. The *β*-actin and 18S primers were used as a standard for control HepG2 cells initially that revealed the same trend with both of the used standards. Then, we used only *β*-actin as a standard reference gene for BLE-treated HepG2 cells.  Figure 5 shows the first decline in gene expression after 48 h of treatment and then increases in the Bcl-2 gene expression after 72 h of BLE treatment with 24 h as a reference gene expression hour for the respective control or treated cells. Gene expression of CDK1 increased and CDK5 and CDK9 showed unusual gene expression during 48 h of treatment. Both CDK5 and CDK9 genes downregulated in expression at 48 h, and after 72 h of treatment, the genetic expression of both of them has increased as compared to the respective hour control. Similarly, CDK10 also absolutely decreased in gene expression after BLE treatment for 48 h and 72 h, as compared to the respective control HepG2 cells (Figure 5).

### 3.6. Phytochemical Analysis of *B. lycium* Constituents by TLC and HPLC

Upon analysis of content by TLC, we found palmatine in all three extracts. Berberine was found at the highest amount in BLE, followed by BA, and a very minute amount was found in the water extract. The retention factor (Rf value) for berberine and palmatine was 0.816 and 0.122, respectively. BLE extract contained 4 more unknown bands at Rf = 0.408 and 0.801, 0.786, and 0.85. To quantify the content of berberine and other phenolic compounds in the BLE extract, we performed quantitative HPLC. Thus, we found berberine at the highest concentration of 48 *μ*g/mL and other phenolic compounds, e.g., benzoic acid was the highest, followed by other compounds in a small amount as listed in Table 2.

## 4. Discussion

Currently, upsurge in cancer cases and inadequate benefits of existing chemotherapies directed us to explore nature for potential herbs against cancer cells [1, 20]. Liver cancer cases are increasing too globally due to the absence of effective therapies; thus, it directed the readers to search for natural and effective strategies that can overcome this rise [3, 20]. Herbal plants have been considered as a vital source of anticancer drugs [21]. However, there is still a great demand for an effective plant extract that can combat chronic disorders, including cancer [22]. Those plants that have been used traditionally are considered as a good source of anticancer agents in reverse pharmacology [23].

*B. lycium* was first defined by John Forbes Royle in 1837 [24]. Its English name is barberry. It is native to the Himalayas regions including India and Pakistan. It has been widely used in folk medicine due to enormous ethnomedicinal properties [25]. It has a broad range of biological activities due to the presence of several identified alkaloids, e.g., berberine, palmatine, and berbamine. We have extracted the roots of *B. lycium* Royle with the solvents of different polarity and screened them against HepG2 cells; thus, we found BLE (50% ethanolic extract) as the most effective extract against HepG2 cells at the IC_50_ value of 47 *μ*g/mL. Besides, we found the highest yield with water (≈236 mg) and the lowest (≈10 mg) with butanol. Our results in terms of yield and cytotoxic activity are justified as different nature of solvents extracted different amount and types of metabolites found in a plant, e.g., water extract usually is enriched in large polysaccharides, proteins, etc., and thus, it gives the highest yield [26], and other solvents extracted various amounts of berberine and other cytotoxic alkaloids/phenolic compounds which is why they exhibited different activity ranges and IC_50_ values [12]. Current findings are supported by the highest amount of berberine and phenolic compounds in a BLE extract as indicated in Table 2 as well as approved by previous studies on other types of cancer cells [12]. We used 50% ethanol-based extract as a result of optimization in our preliminary assays, where we found 50% ethanol as the most effective as compared to 90% ethanol on HepG2 cells by MTT assay. Moreover, the BLE extract has also proven to be selectively cytotoxic to liver cancer cells as depicted by the IC_50_ value of 1.2 mg on HUVECs. This result shows that our extract is more toxic to the liver cancer cell line as compared to normal cells. It is consistent with the hepatoprotective effect of berberine from *B. aristata* used in a study on mice which increased the life span and other hepatoprotective markers of the survived mice [27], and another study used an ethanolic extract of stem bark of *B. Lycium* Royle on mice and it conferred hepatoprotective effects [28].

The plant extracts induce cell death either by apoptosis or by necrosis [29]. The majority of the HepG2 cells underwent apoptosis at low doses, and gradually necrotic cells rose at high doses (Figure 2). The phenomenon of apoptosis is characterized by fragmentation of the plasma membrane and nucleus, thickening of chromatin material, and shrinkage of cells [30, 31]. Our findings are consistent with the apoptosis induction phenomenon in other studies as shown by the sesamol effect on HepG2 cells and also by dandelion water extract on colon cancer cells [16, 32]. The BLE extract exhibited excellent potential against the colony-forming tendency of cancer cells (Figure 2(c)). Thus, it may be a possible mechanism of BLE to weaken the interaction of cancer cells with each other and with the microenvironment as well, which may either prolong or completely dissociate the cancer cell's growth.

The induction of stress environment in cancer cells is one of the causes of cell death [33], as the BLE extract has generated stress by increasing the ROS level inside the HepG2 cells by 1.5-folds at 0.5 mg/mL of BLE treatment, which increased with increase in BLE dose. These findings are consistent with the dose-dependent generation of reactive oxygen species by a dandelion extract [32] and other medicinal plants as well [16, 33]. The generation of reactive oxygen species by treatment with BLE created a stressful environment for cancer cells. As cancer cells are more susceptible to the ROS level as compared to normal cells [33], thus, BLE significantly (60% at 0.25 mg/mL) disrupted the mitochondrial membrane potential resulting in apoptosis of cancer cells. The generation of ROS and changes in mitochondrial membrane potential have also been observed in another study on polyhedral formula against HepG2 cells [34] and also in another study on *Ficus religiosa* extract against breast cancer cells [35].

BLE arrested the HepG2 cell cycle at the S phase at 0.0625 mg/mL. while at 0.5 mg/mL and high concentrations, it accumulated the cells at the G_1_ phase. It may be due to the different behavior of berberine at low and high doses of treatment on the cells, as a study by Khan et al. revealed the arrest of HL-60 cells at the S phase by *B. lycium* Royle crude extract and also berberine [12], while another study on berberine by Yip and Ho on Huh7 revealed the arrest of the cells in the G_1_ phase of the cell cycle [11]. There may be different behaviors of BLE at different concentrations that are worth further studies as some studies on another class of compound, curcumin, indicated its different effects at low and high doses [36].

We studied the effect of BLE extract on certain gene expressions as well. Thus, Figure 5 indicates the decline in gene expression of Bcl-2, p53, CDK5, CDK9, and CDK10 except for CDK1 after 48 h of treatment concerning the reference hour. Tumor-related cell cycle defects are frequently mediated by changes in cyclin-dependent kinase (CDK) activity [37]. It is interesting to see the rise in CDK1 gene expression throughout treatment hours. The degeneration of mitochondrial membrane potential was indicated by downregulation of Bcl-2 gene expression, and downregulation of p53 instead of its upregulation after 48 h and 72 h indicates the p53 independent mitochondrial membrane damage in HepG2 cells by BLE. As CDK1 plays a very crucial role in regulating the cell cycle, its unusual upregulation as compared to other CDKs may be related to its ability to avoid an accumulation of oncogenic mutations during the cell division [37]; however, it needs further detailed studies on BLE-treated HepG2 cells to see the role of interphase and other CDKs. We found BLE as an excellent inhibitor of the liver cancer cell's growth by inducing stress and activating a cascade involving ROS as a second messenger upstream of already known AMPK and JNK pathways [12, 14]; furthermore, it decreased the expression of Bcl-2 that may disturb the Bax/Bcl-2 ratio and completely downregulate mitochondrial membrane potential; it halted the cell cycle at the S and G_1_ phases to stop the replication and cell division among cancer cells.

As a result of the phytochemical analysis, berberine and benzoic acid were found at the highest concentration in BLE extract (Table 2) as a major contributor towards the anticancer effect, along with a possible synergistic role of luteolin and rutin against liver cancer that needs further research. The presence of berberine alkaloids at the highest amount has also been indicated in other studies [12, 24, 38]. However, the phenolic compounds as indicated in our study are characterized by us for the first time according to our knowledge; thus, it needs further detailed study to know the role of these phenolic compounds apart from traditionally known alkaloids.

## 5. Conclusions

This work reports for the first time the anticancer activity of *Berberis lycium* Royle against Human Hepatocarcinoma (HepG2) cells which displayed promising cytotoxic activity against this cell line. These results confirm the evidence that *B. lycium* extracts have an interesting anticancer activity, which could be due to the presence of berberine as the major phenolic compound. The results of this study also provide a scientific basis for the traditional use of *B. lycium* against various diseases including liver cancer.

## Figures and Tables

**Table 1:** The yield and 50% growth inhibition value of different extracts of *B. lycium* Royle roots against HepG2 cancer cells by MTT assay for 48 h.

Solvent type	Yield (mg/1 g of plant)	IC_50_ (mg/mL)
50% ethanol	77.96 ± 0.35	0.047
Methanol	58.56 ± 0.51	0.090
n-Butanol	10.53 ± 0.50	0.088
Acetone	109.33 ± 1.52	0.175
Ethyl acetate	99.06 ± 0.40	0.180
Water	236.5 ± 0.3	0.251
Hexane	79.73 ± 0.64	0.193

**Table 2:** Phytochemical analysis by HPLC revealed the presence of berberine and other phenolic compounds.

Standard compounds	Retention time (min.)	Regression coefficient (*R*^2^)	Conc. in *μ*g/mL in BE extract
Gallic acid	3.008	0.9922	0.237
Rutin	13.22	0.9967	0.372
Benzoic acid	17.959	0.9914	15.81
Quercetin	22.117	0.9992	ND^∗^
Chlorogenic acid	7.201	0.9992	ND
Luteolin	21.8	0.9902	0.399
Resveratrol	19.671	0.9906	ND
*β*-Sitosterol	31.391	0.9964	ND
Apigenin	25.424	0.9962	ND
Palmatine	ND	—	ND
Berberine	18.579	0.990	48.01

^∗^ND: not detected.

## Data Availability

No data were used to support this study.

## Supplementary Materials

Supplementary Materials Supplementary Figure S1: the bar graphs representing % of cells arrested at the S and G_1_ phases by BLE treatment at different doses. Supplementary Table S1: RT-qPCR primers for apoptosis and cell cycle regulatory genes (5′ to 3′).
